# Supplementary material for: Cross-Sectional Exploration of Plasma Biomarkers of Alzheimer’s Disease in Down Syndrome: Early Data from the Longitudinal Investigation for Enhancing Down Syndrome Research (LIFE-DSR) Study
Source: J Clin Med. 2021 Apr 28;10(9):1907. doi: 10.3390/jcm10091907 (PMC8124643; doi:10.3390/jcm10091907)
Supplement: Supplementary file 1 [file jcm-10-01907-s001.zip › jcm-1180511-supplementary.pdf]

Journal of
Clinical Medicine

MDPI

Article

Cross Sectional Exploration of Plasma Biomarkers of Alzheimer's Disease in Down Syndrome: Early Data from the Longitudinal Investigation for Enhancing Down Syndrome Research (LIFE-DSR) Study

Supplemental Materials

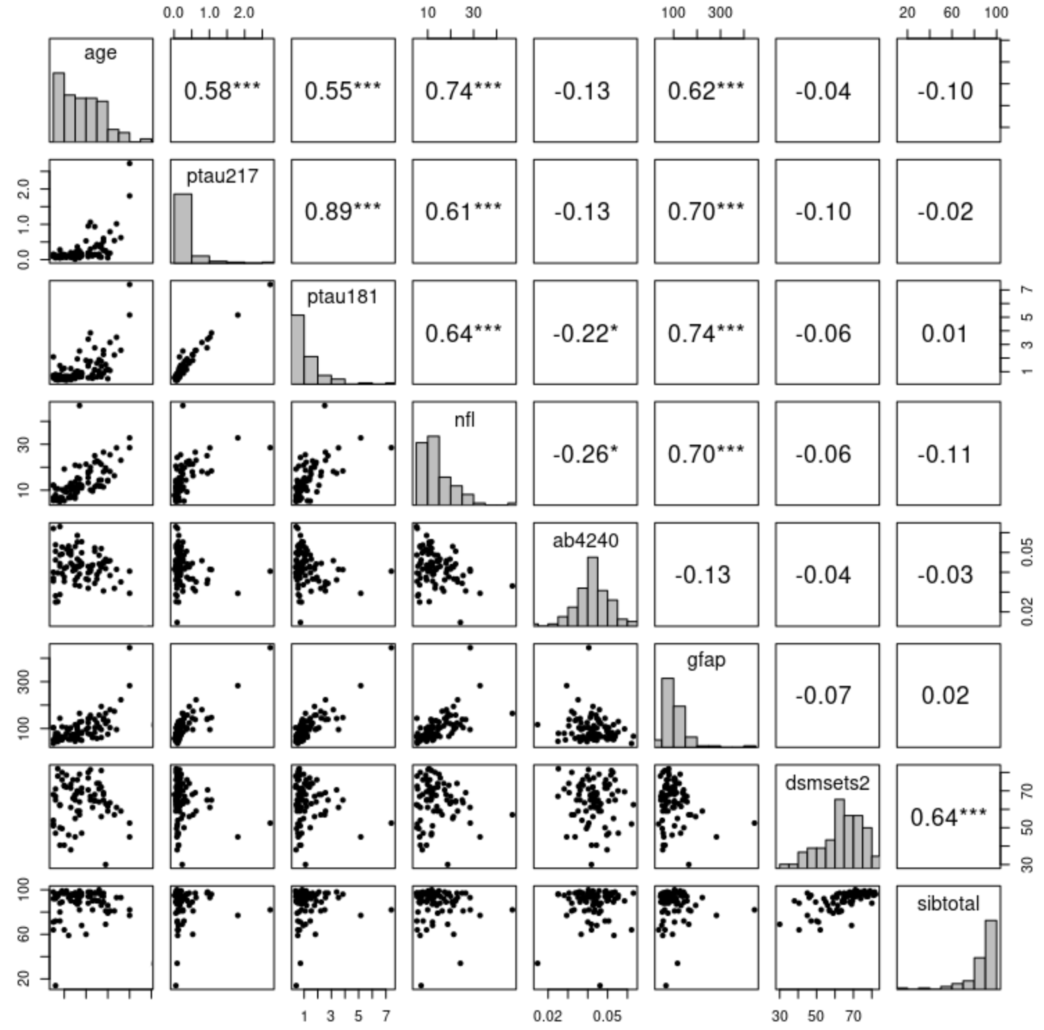

Supplemental Figure 1. Histograms (diagonal), scatterplots (below diagonal), and Spearman correlations (above diagonal) for biomarkers and clinical data. Stars indicate statistical significance at the unadjusted levels of $p \leq 0.05$*, 0.01**, and 0.001***. A Bonferroni corrected significance level is p = 0.05/28 = 0.0018. (Abbreviations: $A\beta_{4240}$ = amyloid $\beta_{42/40}$ ratio, dsmset2 = Down Syndrome Mental Status Examination method 2 score, GFAP = glial fibrillary acidic protein, NfL = neurofilament light, p = p-value, ptau181 = phosphorylated tau at threonine-181, ptau217 = phosphorylated tau at threonine-217, sibtotal = Severe Impairment Battery total score).

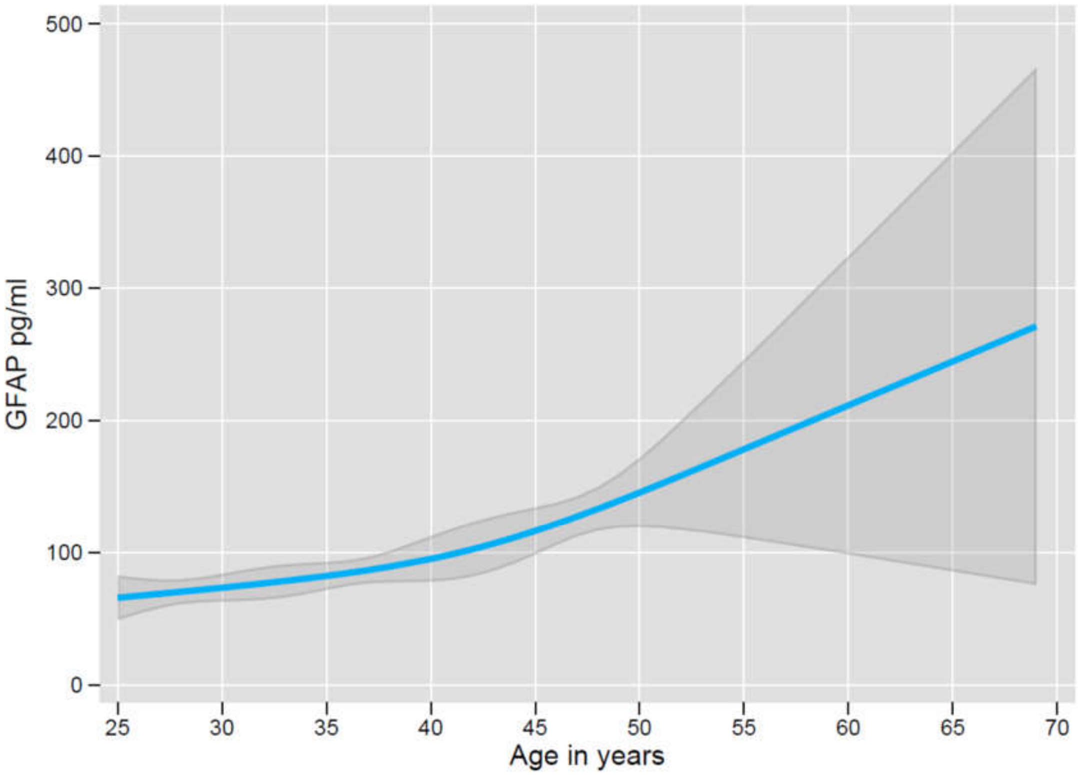

Supplemental Figure 2. Linear regression fit of untransformed GFAP on age, using restricted cubic splines with 4 knots and robust standard errors. F(3,82) = 14.05, p < 0.0001. (Abbreviations: GFAP = glial fibrillary acidic protein, F(df1,df2) = F statistic (degrees of freedom 1, degrees of freedom 2), p = p-value, pg/mL = picogram/milliliter).

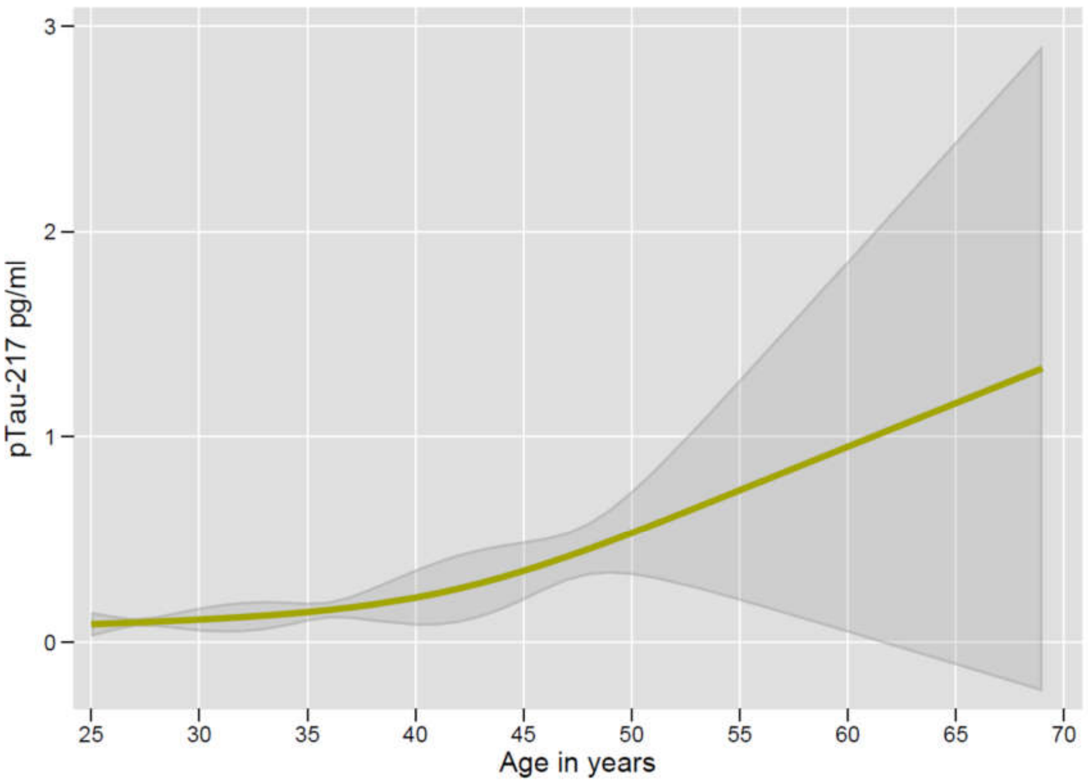

Supplemental Figure 3. Linear regression fit of untransformed p-tau 217 on age, using restricted cubic splines with 4 knots and robust standard errors. $F(3,82) = 9.91$, $p < 0.0001$. (Abbreviations: F(df1,df2) = F statistic (degrees of freedom 1, degrees of freedom 2), p = p-value, p-tau 217 = phosphorylated tau at threonine-217, pg/mL = picogram/milliliter).

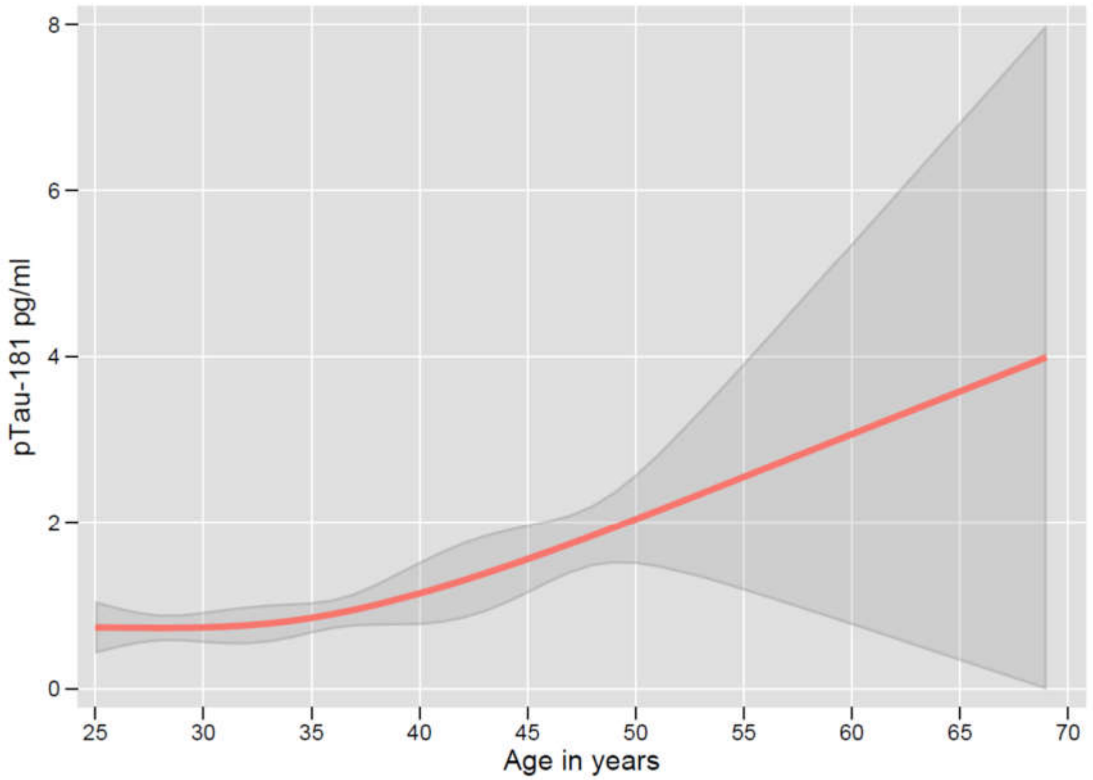

Supplemental Figure 4. Linear regression fit of untransformed p-tau 181 on age, using restricted cubic splines with 4 knots and robust standard errors. F(3,82) = 10.14, p < 0.0001. (Abbreviations: F(df1,df2) = F statistic (degrees of freedom 1, degrees of freedom 2), p = p-value, p-tau 181 = phosphorylated tau at threonine-181, pg/mL = picogram/milliliter).

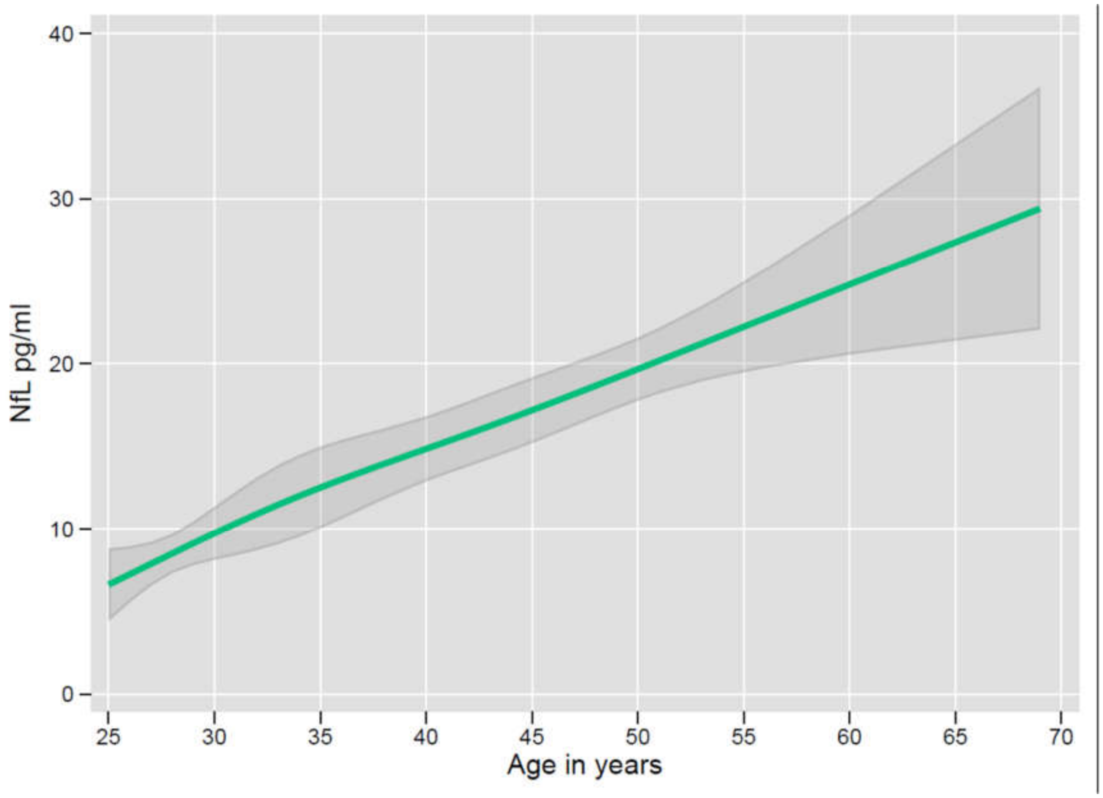

Supplemental Figure 5. Linear regression fit of untransformed NfL on age, using restricted cubic splines with 4 knots and robust standard errors. $F(3,82) = 34.57$, $p < 0.0001$. (Abbreviations: F(df1,df2) = F statistic (degrees of freedom 1, degrees of freedom 2), NfL = neurofilament light, p = p-value, pg/mL = picogram/milliliter).

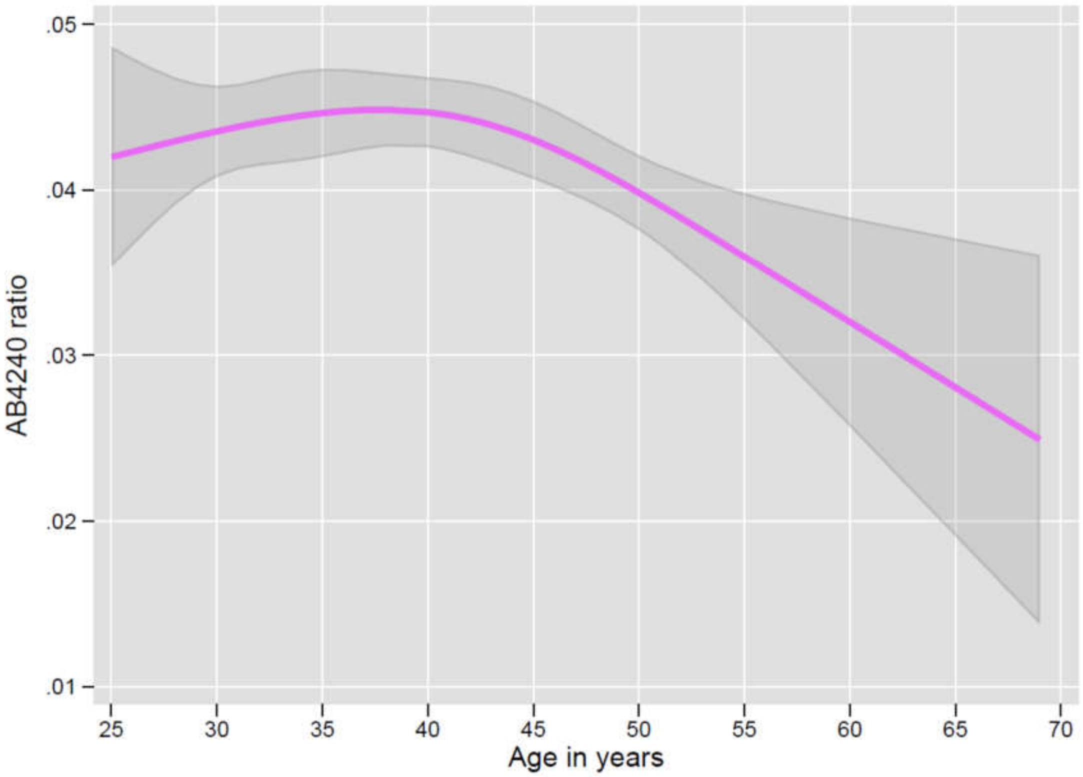

Supplemental Figure 6. Linear regression fit of untransformed $A\beta_{42}/A\beta_{40}$ ratio on age, using restricted cubic splines with 4 knots and robust standard errors. F(3,82) = 4.71, p = 0.0044; F(3,81) = 2.41, p = 0.0728 without oldest individual. (Abbreviations: $A\beta_{4240}$ = amyloid $\beta_{42/40}$, F(df1,df2) = F statistic (degrees of freedom 1, degrees of freedom 2), p = p-value).

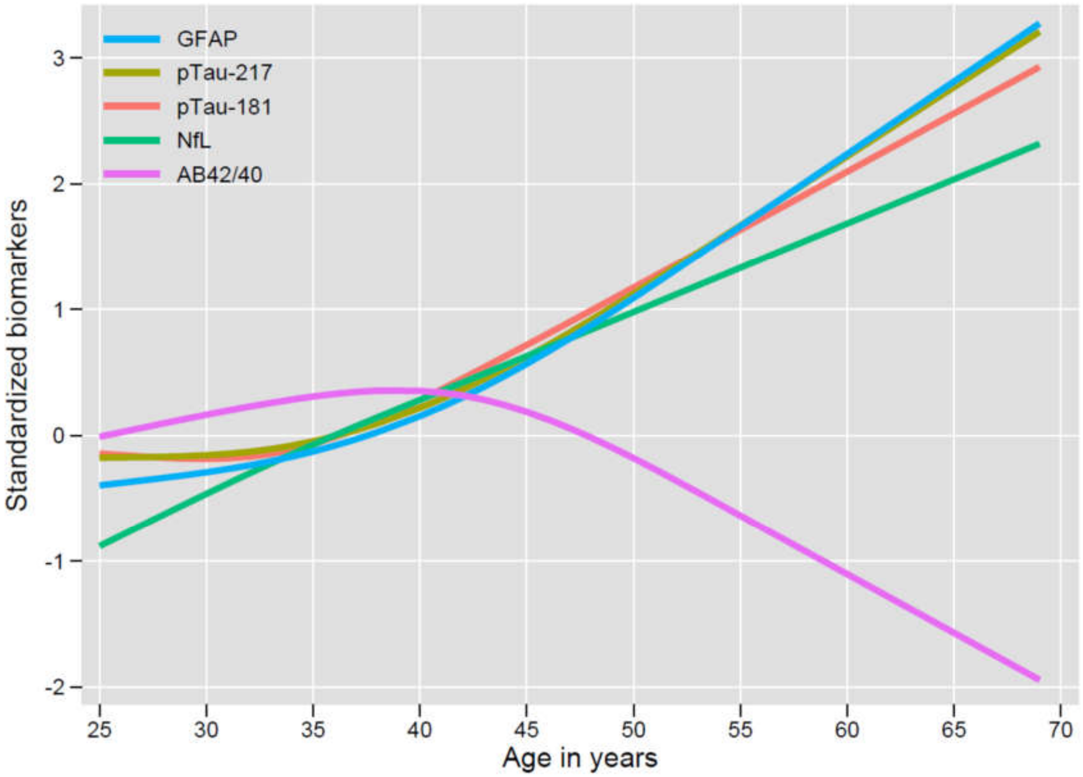

Supplemental Figure 7. Shown are the predictions from linear regressions of each biomarker on age, using restricted cubic splines with 4 knots, and adjusted for *APOE e4* carrier status and sex using additive covariates; *APOE e4* adjusted to non-carrier and sex adjusted to female. GFAP: F(3,80) = 11.23, p < .0001; p-tau 217: F(3,80) = 7.79, p = .0001; p-tau 181: F(3,80) = 9.25, p < .0001; NfL: F(3,80) = 36.02, p < .0001; $A\beta_{42}/A\beta_{40}$ ratio: F(3,80) = 5.01, p = .0031, without oldest individual F(3,79) = 2.04, p = 0.1146. (Abbreviations: $A\beta_{42/40}$ = amyloid $\beta_{42/40}$ ratio, *APOE* ε4 = Apolipoprotein E ε4 allele, GFAP = glial fibrillary acidic protein, F(df1,df2) = F statistic (degrees of freedom 1, degrees of freedom 2), NfL = neurofilament light, p = p-value, p-tau 181 = phosphorylated tau at threonine-181, p-tau 217 = phosphorylated tau at threonine-217).

Publisher's Note: MDPI stays neutral with regard to jurisdictional claims in published maps and institutional affiliations.

Copyright: © 2021 by the authors. Licensee MDPI, Basel, Switzerland. This article is an open access article distributed under the terms and conditions of the Creative Commons Attribution (CC BY) license (http://creativecommons.org/licenses/by/4.0/).

by the author. Licensee MDPI, Basel, Switzerland. This article is an open access article distributed under the terms and conditions of the Creative Commons Attribution (CC BY) license (http://creativecommons.org/licenses/by/4.0/).
